# A novel amyloid MRI imaging based on analysis of texture features representing Aß aggregation

**DOI:** 10.1002/alz.086310

**Published:** 2025-01-09

**Authors:** Yasuko Tatewaki, Hitoshi Yamagata, Benjamin Thyreau, Tetsuya Yoneda, Taizen Nakase, Momoka Asato, Tatsuo Nagasaka, Naoki Kaneko, Jason Hinman, Yasuyuki Taki

**Affiliations:** ^1^ Tohoku University, Sendai, Miyagi Japan; ^2^ Kumamoto University, Kumamoto, Kumamoto Japan; ^3^ Tohoku University Hospital, Sendai, Miyagi Japan; ^4^ University of California Los Angeles, Los Angeles, CA USA

## Abstract

**Background:**

The importance of detecting amyloid β (Aβ) in the early stages of Alzheimer's disease has markedly increased following the approval of Lecanemab, a disease‐modifying drug. MRI is a non‐invasive and less expensive rather than amyloid PET as gold standard for Aβ biomarker, but its clinical ability to detect Aβ has not been demonstrated. MRI phase information reflects paramagnetic substance including iron associated with Aβ aggregation. We have developed a novel approach utilizing multi‐gradient echo (GRE) to perform texture analysis of the phase information, enabling visualization of Aβ. The primary aim of this study is to evaluate the diagnostic capabilities of this "Amyloid MRI" technique.

**Method:**

The consecutive 48 participants (mean age: 72±10, mean MMSE: 25±4) who visited our memory clinic, and underwent both MRI and amyloid PET were enrolled. Amyloid PET images were visually interpreted as Aβ positive (n=30) or negative (n=18), and standardized uptake value ratio (SUVr) maps were generated. Multi‐GRE images were acquired with a 3‐Tesla MRI. We generated a phase slope map, i.e. a first‐order linear estimate of the phase change in time, and segmented four anatomical regions‐of‐interest (ROI) for cortex‐based analysis. Statistical analysis of texture features (mean, SD and entropy) derived from the phase slope map over each ROI was performed. We investigated the correlation between the mean value of regional SUVr and that of texture parameters of the phase slope map, and determined the optimal cut‐off of a linear classifier by ROC analysis based on the diagnosis of amyloid PET.

**Result:**

The correlation coefficients between the mean value of SUVr and the texture features of the phase slope map in parietal, PCCpre, frontal and temporal were 0.57 (p<0.0001), 0.56 (p<0.0001), 0.48 (p<0.0006) and 0.22 (p<0.22), respectively. The optimal cut‐off values for diagnosing Aβ positive or negative showed a sensitivity of 83% (73‐93%), specificity of 89% (74‐103%) and accuracy of 85%(75‐96%).

**Conclusion:**

The texture features of the phase slope map could reflect the spatial aggregation pattern of Aβ. Our method highlighted the potential to visualize Aβ using MRI.

